# SARS-CoV-2 Infection in Wrestlers after International Tournaments, April 2021

**DOI:** 10.3201/eid2911.230278

**Published:** 2023-11

**Authors:** Sakiko Tabata Kuribayashi, Ayu Kasamatsu, Chiaki Ikenoue, Munehisa Fukusumi, Hajime Kamiya, Tomoe Shimada, Teppei Tanaka, Kuniko Murakami, Rieko Takahashi McLellan, Tsuyoshi Sekizuka, Makoto Kuroda, Tomimasa Sunagawa

**Affiliations:** National Institute of Infectious Diseases, Tokyo, Japan (S.T. Kuribayashi, A. Kasamatsu, C. Ikenoue, M. Fukusumi, H. Kamiya, T. Shimada, T. Sekizuka, M. Kuroda, T. Sunagawa);; Japan Wrestling Federation, Tokyo (T. Tanaka);; Ikebukuro Public Health Center, Tokyo (K. Murakami);; Haneda Airport Quarantine Branch, Tokyo (R.T. McLellan)

**Keywords:** COVID-19, HIV, SARS-CoV-2, severe acute respiratory syndrome coronavirus 2, viruses, respiratory infections, zoonoses, wrestling, sports, Japan, Almaty, Kazakhstan

## Abstract

Epidemiologic and genomic investigation of SARS-CoV-2 infections in members of Japan’s national wrestling team after participation in international tournaments in 2021 revealed multiple lineages of SARS-CoV-2 not reported in Japan. The attack rate among wrestlers was high. Results suggest possible transmission during matches. We recommend early case detection and response practices.

Two international wrestling tournaments were held in Almaty, Kazakhstan, during the worldwide COVID-19 pandemic, on April 9–18, 2021: the Wrestling Asian Olympic Games Qualifier and the Wrestling Asian Championships. Approximately 300 athletes from >20 countries participated in those tournaments. On April 14, 2021, SARS-CoV-2 infections were detected in 2 members of Japan’s national wrestling team after returning to Japan. Epidemiologic and genomic investigations were conducted to identify the route of infection. This report was exempt from the requirement for institutional ethics review because it was an epidemiologic investigation approved by the Infectious Diseases Control Law and Quarantine Act in Japan.

The Japanese team comprised 30 wrestlers (29 male and 1 female), 6 sparring partners, and 27 support staff. They stayed in Almaty, Kazakhstan, during April 3–21, 2021. They obtained negative SARS-CoV-2-PCR test results within 72 hours before the arrival at Kazakhstan. The flight between Japan and Kazakhstan was ≈17 hours long. For most of the air trip, the members sat in a block area, maintaining a few seats of distance between the team and other passengers. Team members applied strict precautionary measures; they consistently wore masks and maintained social distance from others during the entire trip. Once in Kazakhstan, their movements were limited to the venue, training site, and designated accommodations. At both tournaments, contact was prohibited with participants from other countries and with team members from other disciplines at both tournaments. Mask use was mandatory for all participants except during matches and training of wrestlers with sparring partners. While in Kazakhstan, the team members tested negative twice by PCR: once upon arrival at their accommodation and then again within 72 hours before leaving for Japan. When they returned to Japan, the members completed mandatory quarantine for 14 days. Quantitative antigen tests were performed on arrival, followed by PCR tests on days 3, 7, and 14.

We defined cases as persons on the Japanese national wrestling team who participated in tournaments in Almaty, Kazakhstan, and were positive for SARS-CoV-2 according to quantitative antigen test, antigen rapid test, or PCR test at the Japanese airport quarantine station or after entering Japan during April 3–May 29, 2021. We obtained epidemiologic and laboratory information from members and the team doctor on the Japanese national wrestling team, as well as from quarantine station officers and officers at the public health center who investigated the cases and their contacts. We performed whole-genome sequencing (WGS) analysis on available isolates at the National Institute of Infectious Diseases, Japan. We performed comparative genomic analysis as described by Sekizuka et al. (*1*). 

A total of 8 cases were reported in 7 wrestlers (7/30, 23%) and 1 staff member (1/27, 4%). No sparring partners on the team tested positive (0/6, 0%). Among the wrestlers, all case-patients experienced symptoms or tested positive within 10 days (median 5 days, interquartile range 2–7 days) after a match day (Figure). Among the 5 wrestlers for whom we obtained screening PCR results, we identified an N501Y mutated strain in 4 cases and other strains in 1 case. Among 3 wrestlers for whom we obtained WGS results, we identified B.1.1.7 lineage in 2 cases and B.1.617.2 lineage in 1 case; WGS also revealed B.1.1.7 lineage in the case in the staff member. None of the 3 B.1.1.7 lineages were identical to any other sequences in our study based on comparative genome analysis (Table). Moreover, none of the sequences had been previously reported in Japan.

**Figure:**
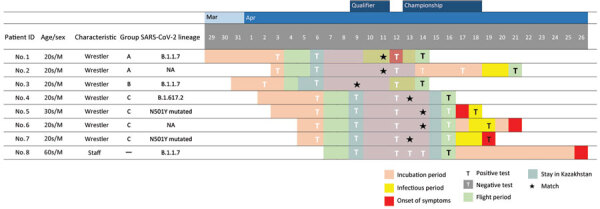
Summary of athlete demographics, SARS-CoV-2 lineages, travel history, clinical course, and testing for 8 SARS-CoV-2–positive cases in the Japanese national wrestling team who attended tournaments in Kazakhstan in April 2021. ID, identification; NA, not applicable.

**Table:** Pairwise nucleotide substitutions in comparative genome analysis of SARS-CoV-2 detected in members of national wrestling team after tournaments, Japan, 2021

Patient ID (GISAID ID)	No. 1	No. 3	No. 8	No. 4
No. 1 (EPI_ISL_1927168)	0	14	14	63
No. 3 (EPI_ISL_1927165)	NA	0	16	63
No. 8 (EPI_ISL_13440457)	NA	NA	0	57
No. 4 (EPI_ISL_1927420)	NA	NA	NA	0

*We sequenced a complete genome from patients 1, 3, and 4 and a draft genome from patient 8. GISAID, https://www.gisaid.org; ID, identification; NA, not applicable.

Our investigation found multiple types of SARS-CoV-2, with >4 types of lineages, including those not previously reported in Japan, in the Japanese wrestling team after participating in international tournaments in Kazakhstan. Although air travel is a possible source of infection (*2*), we suggest transmission likely occurred during the matches. We base our conclusion on the observation of a higher attack rate in the wrestlers (23%), who followed strict precautionary measures, compared with the sparring partners (0/6, 0%), whose activities were very similar to those of the wrestlers except that they did not directly participate in matches. The sport of wrestling has been considered to have the highest risk for transmission of SARS-CoV-2 (*3*,*4*). Beyond a previous study (*5*), our study highlighted the possible transmission of SARS-CoV-2 during the matches, based on the combination of epidemiologic information and WGS results.

Preventing transmission of SARS-CoV-2 in international sports events, particularly high-contact competitions like wrestling, is important to ensure the health of the athletes and prevent export of emerging variants of concern to participants’ home countries, especially those with vulnerable health systems. In this context, monitoring participants in such settings provides an opportunity both to prevent transmission of the virus among participants and for genomic surveillance, using testing for persons with symptoms, swift case isolation, and timely screening for potential contacts before, during, and after events.
